# Children as agents of change in trachoma control

**Published:** 2013

**Authors:** Lisa Dickman, Berhanu Melek

**Affiliations:** Assistant Director, Trachoma Control Program, Health Programs, The Carter Center, Atlanta, USA. Email: lisa.m.dickman@emory.edu; Senior Project Advisor Email: bmelak05@gmail.com

**Figure:**
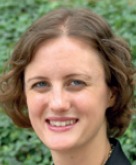
Lisa Dickman

**Figure:**
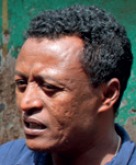
Berhanu Melek

Trachoma is the leading infectious cause of blindness worldwide. Despite the fact that trachoma is preventable, 7.2 million people suffer from trichiasis, the potentially blinding stage of trachoma, and 325 million live in areas where trachoma is confirmed or suspected. According to the World Health Organization, 21 million people have trachoma (Weekly Epidemiological Record No. 17, 27 April 2012).

This situation requires immediate action if the global target to eliminate blinding trachoma by 2020 is to be reached.

The WHO endorses the SAFE strategy to prevent and treat trachoma. **S**urgery treats trichiasis patients, **A**ntibiotic distribution treats active infections, and **F**acial cleanliness and **E**nvironmental sanitation prevent the transmission of trachoma.

In Ethiopia, the country with the heaviest known burden of trachoma cases in the world, health educators promote the SAFE strategy in a range of different settings.

Schools are an ideal place to target children, who are most susceptible to trachoma infection. In 2005, the Carter Center began health education in 700 schools in the Amhara National Regional State. Teacher training was scaled up in 2008 and 2009, following the expansion of the programme to cover the entire region. In total, 7,822 primary schools now have ongoing health education.

The Carter Center trained one science teacher and the director from each primary school to implement the trachoma primary school curriculum developed jointly by The Carter Center and the ministry of health. In a recent assessment of school-based activities, we found that school children supported the SAFE strategy in several key ways.

- **Children can help to identify family members who have trichiasis**. Teachers instructed children to go home and ask whether any family member had ‘hair in the eye.’ Children asked their family members if they knew what caused the condition. Common myths included that trichiasis was the result of a curse or the fault of the individual.
- **Children can educate family members about trichiasis**. The children, empowered with information from their teacher, explained that trichiasis is caused by a disease and is not their fault. Children's views were generally respected by their family, who were proud to have a child who is attending school.
- **Children can support mass drug administration campaigns**. They can do so by participating themselves and by encouraging family members to take azithromycin.
- **Children can learn improved hygiene habits**. In schools where water is provided and where there is sufficient health education and mobilisation, children can learn to practice good facial cleanliness and help to monitor the facial cleanliness of other children. In addition, they can care for younger siblings and can be encouraged to clean their faces for them.
- **Improved sanitation**. At school, children learn how to use latrines and how these latrines can prevent diseases like trachoma. Health clubs at some schools teach students about healthier hygiene habits and how to prevent trachoma. Health, trachoma, and environmental sanitation clubs help to organise environmental sanitation campaigns at school and in the community. We also met children who had convinced their families to construct latrines at home.

Healthy habits that children learn in school improve their health and the health of their families and future generations. Children in secondary schools are particularly effective at educating families and encouraging behavioural change in their families, such as keeping faces clean and using latrines. Older school children are the parents of the immediate future who will raise their families with healthier habits and they should receive targeted trachoma health education. For all children, school-based health education in addition to other form's of health education – such as radio messages, health extension workers, and social organisations – collectively work to demystify trachoma, reduce stigma and shame, and to prevent blindness.

**Case Study: Iyasta Primary School**

The students and staff of Iyasta Primary School in Ankesha Woreda district played a unique role in promoting health education and the SAFE strategy in their community. In this district, people have resisted taking azithromycin during the twice-yearly MalTra Week campain. This campaign distributes eye ointment containing azithromycin and tetracycline to half of the people in the Amhara National Regional State in Ethiopia in May, and to the rest in November. Primary school children, themselves initially reluctant to take the drug, were educated by the local health extension workers about the transmission, progression, and prevention of trachoma. The children then encouraged their parents to take the drug, resulting in a 60.4percnt; increase in coverage compared with the previous year. People noticed that the signs and symptoms of trachoma and other diseases resolved after taking the drug and now request azithromycin.

**Figure:**
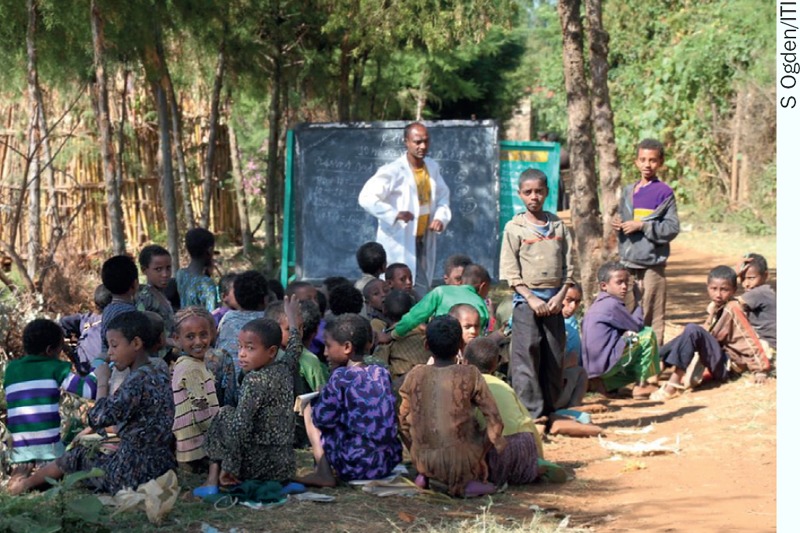
Students learn both in classrooms and outdoors when there is insufficient classroom space. ETHIOPIA

The following conditions supported the success of mobilising students to assist with the MalTra campaign.

- Routine supervision and a post-MalTra Week review meeting identified that the area had low coverage in time to intervene before the next campaign.
- The health workers in the area already had a relationship with the school and were motivated to go and speak with the school staff.
- School staff were willing to assist, and allowed class time for health extension workers to speak with the children.
- The health education delivered by the health extension workers was effective enough to convince children to take the drug and speak to their families.

